# The advantage of sex: Reinserting fluctuating selection in the pluralist approach

**DOI:** 10.1371/journal.pone.0272134

**Published:** 2022-08-02

**Authors:** Jean-Sébastien Pierre, Solenn Stoeckel, Eric Wajnberg

**Affiliations:** 1 UMR 6553 Ecologie Biodiversité Evolution, CNRS INEE, Université de Rennes 1, OSUR, Campus de Beaulieu, Rennes Cedex, France; 2 IGEPP, INRAE, Institut Agro, Université de Rennes, Le Rheu, France; 3 INRAE, Sophia Antipolis Cedex, France; 4 Projet Hephaistos, INRIA, Sophia Antipolis Cedex, France; Hebrew University of Jerusalem, ISRAEL

## Abstract

The advantage of sex, and its fixation in some clades and species all over the eukaryote tree of life, is considered an evolutionary enigma, especially regarding its assumed two-fold cost. Several likely hypotheses have been proposed such as (1) a better response to the negative frequency-dependent selection imposed by the “Red Queen” hypothesis; (2) the competition between siblings induced by the Tangled Bank hypothesis; (3) the existence of genetic and of (4) ecological factors that can diminish the cost of sex to less than the standard assumed two-fold; and (5) a better maintenance of genetic diversity and its resulting phenotypic variation, providing a selective advantage in randomly fluctuating environments. While these hypotheses have mostly been studied separately, they can also act simultaneously. This was advocated by several studies which presented a pluralist point of view. Only three among the five causes cited above were considered yet in such a framework: the Red Queen hypothesis, the Tangled Bank and the genetic factors lowering the cost of sex. We thus simulated the evolution of a finite mutating population undergoing negative frequency-dependent selection on phenotypes and a two-fold (or less) cost of sexuality, experiencing randomly fluctuating selection along generations. The individuals inherited their reproductive modes, either clonal or sexual. We found that exclusive sexuality begins to fix in populations exposed to environmental variation that exceeds the width of one ecological niche (twice the standard deviation of a Gaussian response to environment). This threshold was lowered by increasing negative frequency-dependent selection and when reducing the two-fold cost of sex. It contributes advocating that the different processes involved in a short-term advantage of sex and recombination can act in combination to favor the fixation of sexual reproduction in populations.

## Introduction

The maintenance of sexual reproduction and its fixation in some eukaryote species is often described as a puzzle in evolutionary biology, even in recent literature reviews [1–5]. If most eukaryotes reproduce using both clonal (also known as asexual reproduction, including parthenogenesis and apomixis) and sexual reproduction, a significant number of species and of populations within species has lost the possibility to reproduce clonally and now exclusively reproduce through sexual reproduction, significantly affecting their ecology, genetic and evolution [6–10]. Sexuality is an ancient and ubiquitous reproductive mode in eukaryotes [11] but clonal forms have a potential two-fold advantage against sexuality by avoiding the cost of producing males and are supposed to quickly win the competition over a few generations [12, 13]. However, several arguments explain the maintenance and fixation of sexual reproduction [1, 4, 14–27]. Considered one by one, these arguments are now convincing enough from a theoretical point of view to explain some specific experimental observations, but their relative importance is not yet fully assessed [27]. Several authors pleaded also for a so-called pluralist approach [5, 28, 29], arguing that the different explanations are likely to act synergistically.

Hypotheses explaining the advantage of sex fall into one of five main groups: (1) the Red Queen hypothesis inducing negative frequency-dependent selection on genotypes, (2) the Tangled Bank hypothesis, relying on local competition among siblings (its effect is coined as density-dependent but is likely to result in a negative frequency-dependent selection), (3) the genetic consequences of recombination, (4) ecological factors reducing the benefit of high fecundity in the framework of *K* selection and (5) randomly fluctuating selection over time. Hypotheses (1) and (3) have been intensely studied, while hypotheses (4) and (5) have received far less attention.

The first hypothesis, named the frequency-dependent selection hypothesis [30], more or less time-lagged, as expected in the Red Queen coevolution hypothesis (also referred as the chase Red Queen and the Red Queen dynamics) [14, 16, 18, 19, 24, 31–33], considers that, as a result of the coevolution between hosts and their pathogens (or prey and their predators, or hosts and their parasitoids), a genotype is more likely to be attacked when it is more frequent in the population and thus the fitness of genotypes (and their resulting phenotypes) increases as their frequency in population decreases (subjecting genotypes to evolve under negative frequency-dependent selection due to co-evolution between biological antagonists). From a quantitative genetic standpoint, sex increases the genotypic variance in the host progeny, offering less adaptive opportunities to biological antagonists. In the second hypothesis, named the Tangled Bank hypothesis, monomorphic siblings suffer a strong local competition favoring the genotypes more different which can occupy slightly different niches. This last hypothesis was advocated as too early dismissed by [34] and formalized by [35]. It is likely to result also in negative frequency-dependent selection [36], but on a spatial rather than temporal basis.

The third hypothesis assumes genetic advantages of sex over clonality and is often named the Fisher–Muller theory of sex [37–39]. Sex involves recombination, which results in the random partition of deleterious and beneficial mutations into different descendants on which selection may act to favor lines with fewer deleterious mutations and more beneficial ones [37, 38, 40]. Therefore, the genetic load resulting from weakly deleterious mutations is thereby better controlled or even eliminated in sexual organisms, while clonal forms with less recombination accumulate mutations that can likely be slightly deleterious on expressed genes. The deterministic and stochastic accumulation of weakly detrimental mutations on clonal genomes, respectively known as Mutational Deterministic Hypothesis [17, 41] and Muller’s ratchet [42], has been considered to be too slow to challenge the two-fold advantage of clonal reproduction [27]. In clonal eukaryotes with large-sized genomes, accumulated mutations may have an overall synergistic epistatic negative effect on fitness, which would lower the clonal advantage to much less than two-fold [27, 43]. Yet, experimental evidence for the negative effects of synergistic epistasis among deleterious mutations is equivocal [43, 44], suggesting that it would be relatively uncommon in real populations [43].

The fourth hypothesis, addressed by few authors [23, 45], considers the realistic ecological conditions in which the competition between sexual and clonal variants occurs. In the context of *K* selection [46], the two-fold advantage of clonality diminishes as the intensity of intraspecific competition increases. Here investment in offspring to ensure their competitive ability in the acquisition of resources is more valuable than the production of more offspring. This hypothesis results, fundamentally, in a reduction of the two-fold advantage.

Finally, the fifth hypothesis addresses the question of random fluctuating selection. Sexual recombination can compensate the uncertainty in the direction of future selection due to unpredictable stochastic environmental fluctuations. Under such conditions [47, 48], proposed the first theoretical explanation for the benefit of genetic polymorphism, of which sex can be seen as a special case. Sexual reproduction can preserve a large amount of variation in allele combinations within genomes, while clonality rapidly leads to limited combinations of alleles and even one genome in finite populations, under dominant genetic drift or directional selection [10]. In this framework, sexual reproduction appears as a form of a bet-hedging and also as a risk-averse strategy [49, 50]. Bet-hedging is a strategy which consists in splitting bets over several targets instead of one [51, 52]. The advantage of sexual reproduction under fluctuating selection has been thoroughly studied by quantitative geneticists [20, 53–55]. In their seminal works [20], after [56], confirmed the existence of a minimal level of fluctuating selection needed to ensure an advantage to recombination sufficient to overcome the two-fold cost of sex.

### Combined effects

Each of the mechanisms for the advantage of sex are usually presented independently and discussed as if they were mutually exclusive, yet their effects are likely to act either additively or interactively., A review in 1999 strongly advocated in favor of a combined study, taking the example of the interaction between the Red Queen hypothesis and the mutational hypotheses [57, 58]. This was previously attempted by [9, 29], and later by [5]. Recently [5], wrote a representative review in which the incorporation of the random fluctuating selection hypothesis was mentioned but not discussed in depth. We therefore adopted a pluralistic point of view by considering the simultaneous effect of two processes favoring variance, *i*.*e*., randomly fluctuating environmental and negative frequency-dependent selections, in order to investigate whether their combined action may counter-balance the theoretical two-fold disadvantage of sex. Finally, we studied the effect of the fitness of clonal forms, simply by reducing their basic advantage (relative fitness; in constant environments) to a factor lower than two, and examined the consequences for the conditions needed to ensure the fixation of sex.

## Description of the model

To identify the conditions under which sexual reproduction outcompetes clonal reproduction, we developed a Monte Carlo simulation model tracking the evolution of haploid individuals with inherited reproductive mode (sexual or clonal) in non-overlapping generations, in finite mutating populations undergoing temporal random fluctuation of selection acting on their phenotypes. Simulations all began with 2000 sexual and 2000 clonal individuals and population size was maintained constant over generations until one reproductive mode was fixed. All individuals inherited their reproductive mode from their parents.

Genotypes of individuals were simulated as haploid chromosomes, each of them represented by a string of 50 binary genes coding for either 0 or 1. The phenotype of each individual was considered to be the sum of its allele values, thus ranging from 0 to 50. Thereby, different genotypes would result in the same phenotype if the sums were the same. At the beginning of each simulation, alleles at each gene were drawn randomly with a probability of 0.5, so that the expected mean and variance of phenotypes in the entire population were 25.0 and 12.5, respectively. Per generation, and before offspring production, population at each locus mutated at a rate of μ = 5.10^−4^ (*i*.*e*., two allele mutations per gene per generation overall the population to avoid gene fixation by genetic drift in finite population, 0.025 mutation per genome per generation, which matches observed mutation rates [59–61]. Our results therefore can be directly compared to those obtained by [20]. In addition, to include the frequency-dependent selection hypothesis [30], we added a frequency-dependent mortality process with no time lag. It models situations where there is an advantage to be genetically different from the majority type, like when ‘predators’ or ‘parasites’ adapt to the evolving phenotypes of the studied population and constitutes a first perfectible approach to tackle the putative effects of a Red Queen dynamics. At each generation, mortality rates of each phenotype before reproduction were proportional to their frequency within the population.

The fitness of each individual in each generation depends on its phenotype *x* and on the characteristics of its environment. Following [19], fitness was computed using the following equations:

For a clonal individual:

$${F_{i}=e}^{-\frac{{\left(x-{\theta_{i}}^{*}\right)}^{2}}{2\omega^{2}}}$$ (1)

For a sexual individual:

$$F_{i}={\frac{1}{b}e}^{-\frac{{\left(x-{\theta_{i}}^{*}\right)}^{2}}{2\omega^{2}}}$$ (2)

When *b* = 2, the cost of sexual reproduction is two-fold [12]. We also tested different values for this parameter, *i*.*e*., 1.8, 1.6, and 1.2.

*θ*_i_* is the environmental condition under which the average fitness of the population is maximal at generation *i*, and *ω*^2^ is the inverse of the selection strength. By definition, in our model, 2*ω* ecologically corresponds to the width of one environmental niche. All computations were run by fixing *ω* = 4.0, settling the width of an ecological niche to a value of 8.

Individuals with the higher fitness have a higher chance to contribute to the next generation. Hence, individuals contributing to the next generation were drawn, with replacement, using a probability proportional to their fitness. Also, individuals descending from clonal lines each produced two progenies carrying the same haplotype, identical (except for mutation differences among offspring) to their single parent. Individuals descending from sexual lines needed at least another sexual mate in the population to produce two descendants. In this case, two parents were randomly drawn from the sexual pool to produce two possibly recombined offspring. The probability that one crossing-over event recombined the parental haplotypes was fixed at 0.75 per generation in all the following computations. The location of crossing over events was random along the chromosome. To see whether environmental variability can have an influence on the long-term success of the two modes of reproduction, simulations were done by randomly drawing, in each generation, the value of θ*, *i*.*e*., the environmental condition in which the average fitness is maximal, from a Normal distribution with mean 25.0 and different standard deviations, ranging from 0.0 to 30.0, in steps of 0.5. The environmental conditions may thus fall outside the range of the possible phenotypes, but, as our model simulated soft selection, *i*.*e*., selection acts relatively to phenotypes in a constant population size, in this way, it acted homogeneously on population over generations. Standard deviation values were drawn either independently at each generation (no autocorrelation) or with an autocorrelation of 0.6 to assess whether predictability in environmental fluctuation may temper the overall success of sexual reproduction. To assess if negative frequency-dependent selection as a proxy for both the Red Queen and potentially Tangled Bank hypothesis may influence the outcome of competition between sexual and clonal lines, we added, before drawing individuals contributing to the next generation and thus before offspring production, a frequency-dependent mortality process. In each generation, and before selection, each individual may die before reproducing at a rate proportional to *P*_*i*_/*m*, where *P*_*i*_ is the frequency of the *i*^th^ phenotypic class the individual belongs to, and *m* is a constant describing the intensity of frequency-dependent mortality. Six different values of *m* (*i*.*e*., 1, 2, 10, 20, 100, and infinity) were compared; *m* = infinity corresponds to the absence of frequency-dependent mortality. Finally, additional simulations were run by reducing the two-fold cost of sexual reproduction (parameter *b* in Eq 2) to 1.8, 1.6, and 1.2 to simulate the effect of an increased genetic load due to an overall synergistic epistatic negative effect of mutations on fitness in clonal individuals [17, 21, 62, 63] or fitness limited by intra-specific competition [23]. To compare our results with the predictions of [20, 56] about the level of environmental variability needed to select for sexuality with recombination, we also explored the effect of reducing the cost to 1, which means no cost of sexual reproduction. In that case, the only difference between sexual and clonal types was recombination.

Each set of conditions was simulated using 100 independent repetitions and we studied, for each set of conditions, the proportion of replicates in which sex was fixed. We tracked conditions when simulations where sex began to fix as the exclusive reproductive mode of a population. This proportion always increased from zero to one as the magnitude of environmental fluctuations increased, showing generally a logistic curve. To compare quantitative effects, we computed for each parameter set the point at which sex was fixed in 50% of the populations as the inflection point of a fitted logistic regression along environmental fluctuations in R [64].

To compare the intensity of selection in fluctuating environment and its range of changes endured by our simulated populations fixing sexual and clonal reproduction, and also to compare it to the intensity of selection monitored in natural populations, we computed the selective coefficient endured by the population for each simulation over generations as $S_{i}=\frac{{\bar{w}}_{i}-W\theta_{i}}{W\theta_{i}}$, following [65, 66]. In this formula, *Wθ*_*i*_ corresponds to the best possible fitness of the most adapted phenotype *θ*_*i*_ to the environmental conditions at generation *i* while ${\bar{w}}_{i}$ is the mean fitness of all the phenotypes in the population at this generation. We set the reference fitness as the fitness corresponding to the best adapted phenotype at one generation. The mean fitness of the selected population is lower or, at most, equal to this value. This results in negative, or at best zero, selective coefficients.

We also computed the number of generations needed to fix one of the reproductive modes, variations of selective coefficients endured by population and the variance of phenotypic values (hereafter, phenotypic variations) along two consecutive generations. We reported these values in results as mean ± standard deviation over all simulations.

## Results

**Fig 1:**
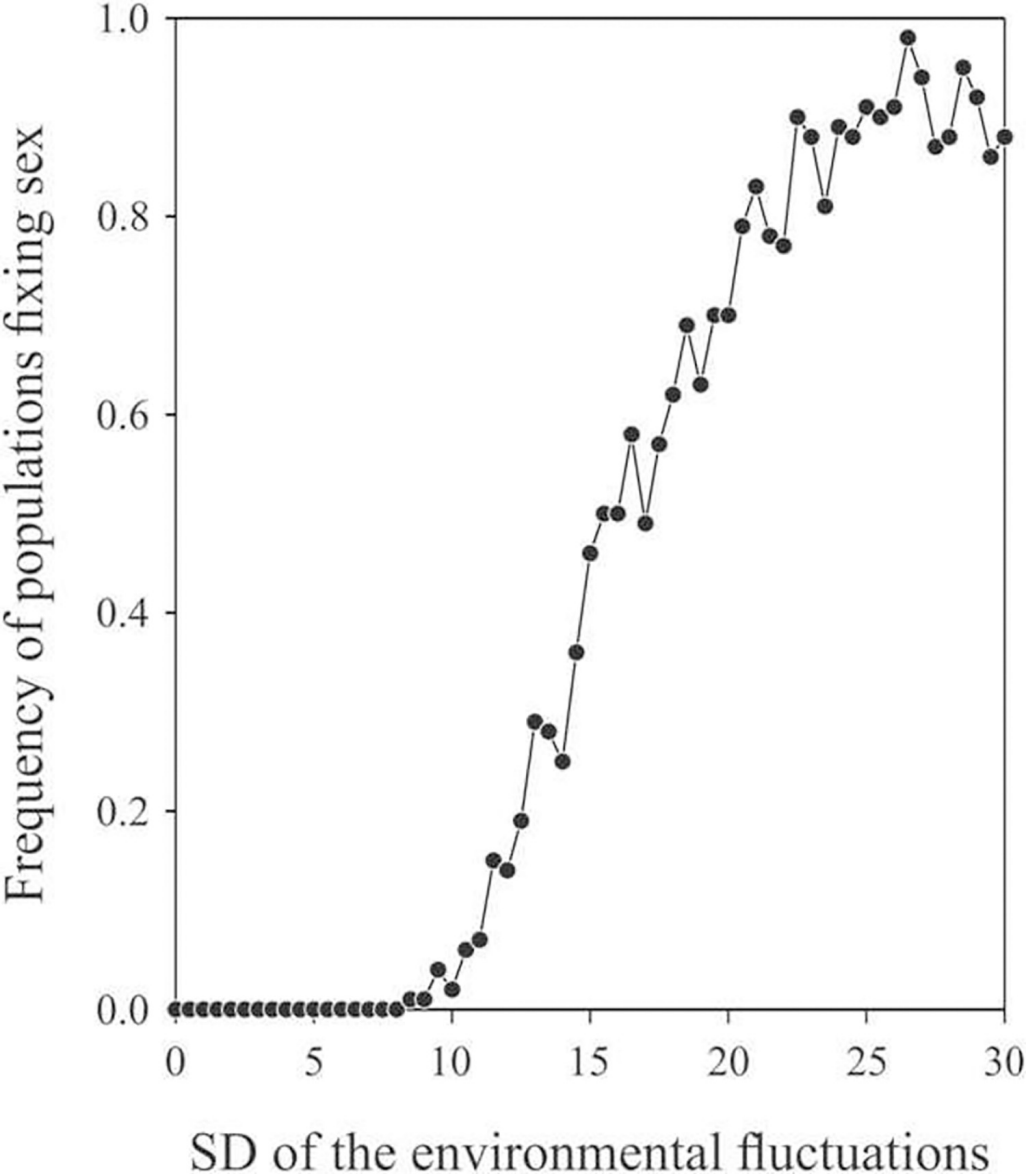
Proportion of populations where sexual reproduction fixed as a function of the variance of random environmental fluctuations. In this example, a SD of 8 resulted in the fixation of sex in more than 5% of the populations, corresponding to the case with no negative frequency-dependent selection resulting from the Red Queen Hypothesis and with no inter-generational autocorrelation in environmental fluctuations. In these simulations, ecological niche width was defined as 2*ω* = 8.

Increasing environmental fluctuation progressively leads to an increase in the proportion of populations fixed for sexual reproduction (see one example in Fig 1). Fixation of sexual reproduction in populations began to occur when standard deviations of environmental fluctuations were higher than 8. Under a standard deviation of environmental fluctuations of 8, sexuality was fixed in less than 25 generations (and more than 5; mean: 12.7 ± 4.5; over 100 simulations that fixed sexuality) while clonality was fixed in less than 29 generations (mean: 14.0±4.2; over 100 simulations that fixed clonality). In populations that fixed sexuality, selective coefficients ranged from -0.062 to -1.000 (mean: -0.676 ± 0.315) while selective coefficients endured by populations that fixed clonality ranged from -0.017 to -1.000 (mean: -0.571 ± 0.337). Populations that fixed sexuality presented mean values of phenotypic variations of 6.3 ± 2.5 while population that fixed clonality showed twice less phenotypic variations (mean: 3.4 ± 3.1).

Fifty percent of the simulated populations fixed sexual reproduction when random environmental fluctuations reached 17.93 ± 0.15. In this case, sexuality begin to fix in populations from only three generations (mean: 10.9 ± 5.9; over 100 simulations that fixed sexuality) while enduring selective coefficients from -0.044 to -1.000 (mean: -0.806 ± 0.288). Again, population that fixed sexuality presented nearly twice the phenotypic variations (mean: 5.8 ± 2.8) of populations that fixed clonality (mean: 2.3 ± 3.1).

**Fig 2:**
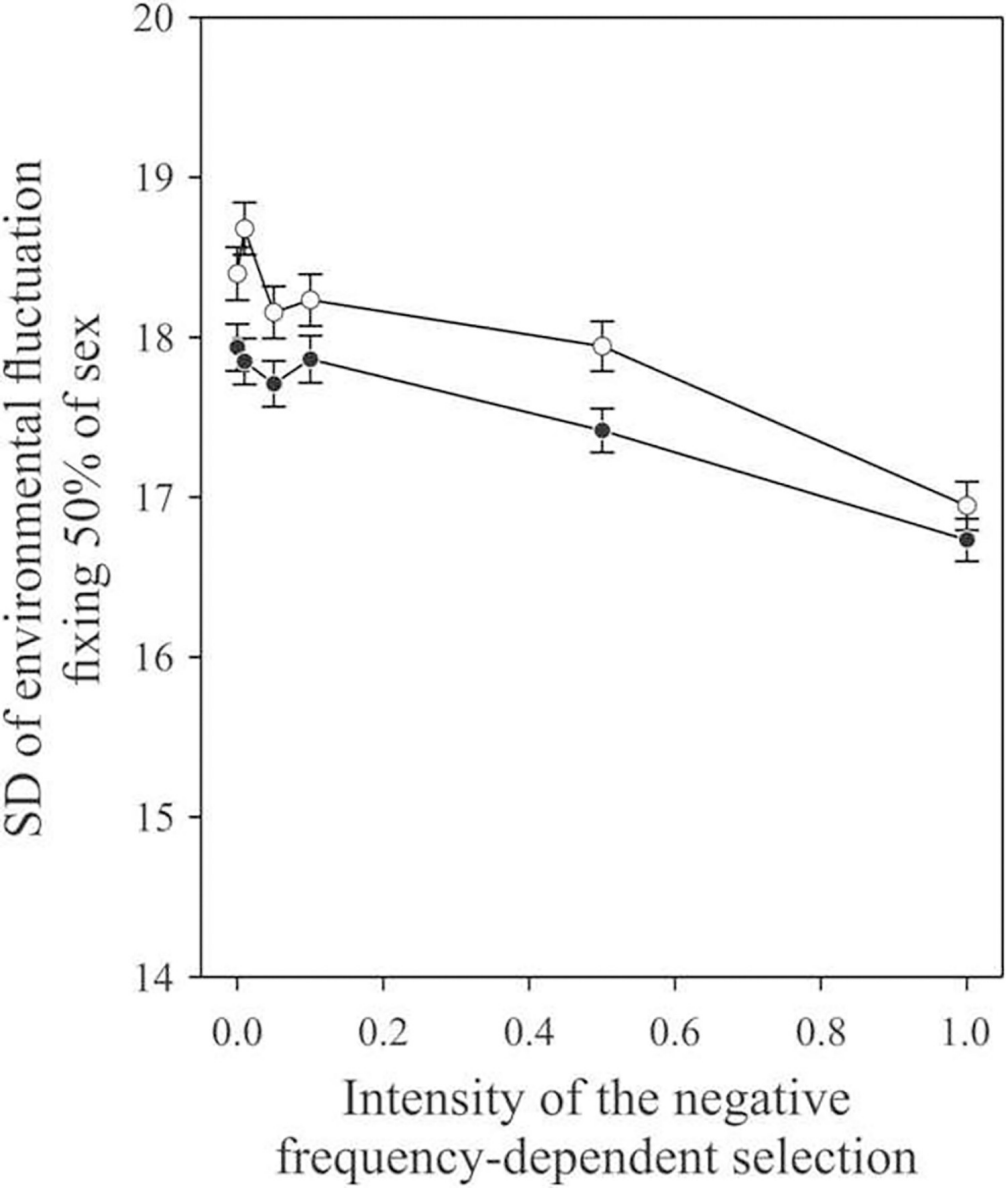
Standard deviation (± SE) of random environmental fluctuations resulting in the fixation of sex in more than 50% of the populations. Results are shown as a function of the negative frequency-dependent selection resulting from the Red Queen Hypothesis (inverse of parameter *m*) and inter-generational autocorrelation in environmental fluctuations. Closed dots: uncorrelated fluctuations, Open dots: auto-correlated fluctuations of 0.6.

Adding negative frequency-dependent selection decreased the amount of environmental fluctuation needed to fix the same proportion of populations for sexual reproduction (Fig 2). In all cases, the probability that populations would become fixed for sexual reproduction increased more with increasing environmental fluctuation than with frequency-dependent selection. Finally, adding inter-generational autocorrelation in environmental fluctuations increases the standard deviation needed to reach the switching point (see Fig 2). This effect is highly significant and can counterbalance almost exactly an intensity of negative frequency-dependent selection of 0.5 (m = 2). Such effects are discussed in the Discussion part below.

**Fig 3:**
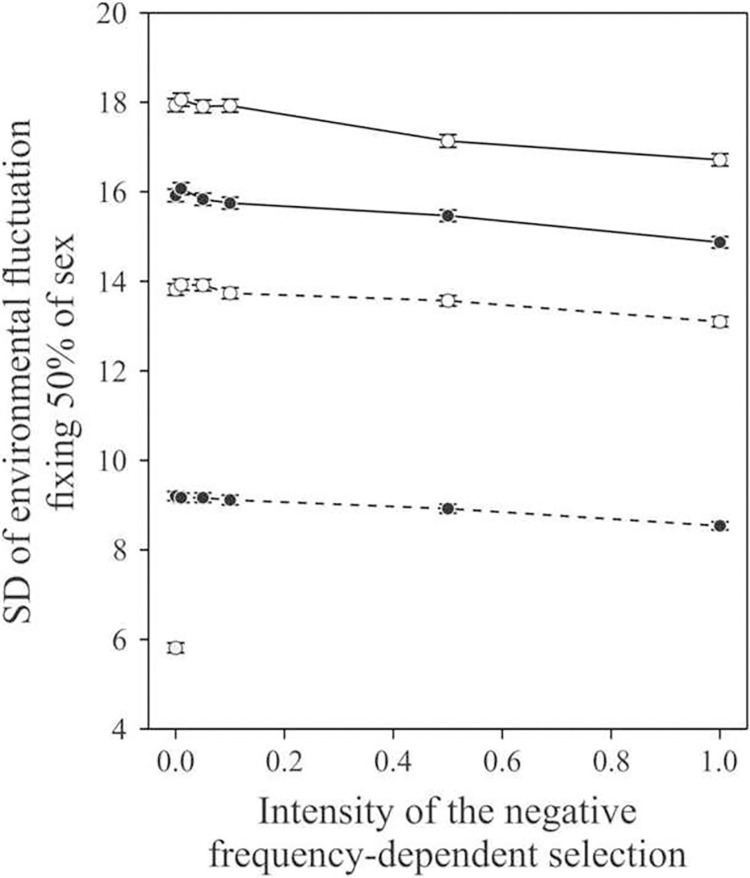
Standard deviation (± SE) of random environmental fluctuations resulting in the fixation of sex in more than 50% of the populations. Results are shown as a as a function of the intensity of the negative frequency-dependent selection resulting from the Red Queen Hypothesis (inverse of parameter *m*) and for different levels of the cost of sex. Open dots and solid lines: cost of sexual reproduction of 2.0; closed dots and solid lines: cost of sexual reproduction of 1.8; open dots and dashed lines: cost of sexual reproduction of 1.6; closed dots and dashed lines: cost of sexual reproduction of 1.2. The single point in the lower left part of the plot corresponds to no cost of sex and no negative frequency-dependent selection, allowing comparison with the previous prediction of [54] (see text).

We then considered the combined effect of environmental fluctuation, negative frequency-dependent selection and the reduction in fitness of clonal individuals (Fig 3). In all combined sets of parameters we explored, a sufficient amount of environmental fluctuations always allowed for the fixation of sexual reproduction. The amount of random environmental fluctuations needed to fix sexual reproduction in 50% of the simulated populations was only moderately reduced from 17.93(SE = 0.15) to 16.71 (SE = 0.13) which represents a decrease of 6.80% (SE 0.011) by negative frequency-dependent selection, even at its maximal strength. Decreasing the cost of sex from 2.0 to 1.6 alone reduced the inflection point from 17.94 (SE = 0.15) to 13.82 (SE = 0.13). Reducing the cost of sex to 1.2 combined with a maximal strength of negative frequency-dependent selection reduced the magnitude of random environmental fluctuations needed for sex to become fixed in 50% of the populations by a factor of approximately 2 (1.948 exactly), from 17.93 (SE = 0.15) to 9.70 (SE = 0.103). Finally, for the range of environmental variation we studied, the effects of the strength of negative frequency-dependent selection varied nearly linearly with the advantage of clonal reproduction. A multiple regression on the value of the inflection points against the cost of sex and the strength of inverse negative frequency dependent selection (1/*m*) resulted in a very good fit (adjusted R^2^ = 0.9983). We found no evidence of curvature and no interaction, implying that these two factors are linear and additive. Finally, we also computed the case where there is no cost of sex (*b* = 1 instead of 2) resulting in a lower inflection point of 5.81 (the isolated point on the bottom left of Fig 3).

## Discussion

Our results, using an individual-based model simulating the competition between sexual and clonal reproduction, argue that random environmental fluctuations may play an underestimated role in the advantage and fixation of sex in populations. Sexual reproduction began to fix in populations when the standard deviation of the random environmental fluctuation exceeded 8 without autocorrelation and negative frequency-dependent selection, and with a standard two-fold cost of sexual reproduction. This value corresponds to the width of an environmental niche in our simulations (defined as 2*ω* with *ω* set to a value of 4 in our model). Similar environmental variations were already observed and measured, for example concerning monthly variations of concentrations of different heavy metals in natural aquatic sediments along rivers and coasts monitored over up to seven years [67]. In our model, these environmental variations caused temporal variations of selective coefficients over the years very similar to those measured in some snails, birds, fishes and plants populations [68–70]. In addition, our simulations demonstrated that such environmental variations only had to apply over 5 to maximum 25 generations to fix sexuality in more than 5% of the populations. The point where 50% of the populations became fixed for sex happened when the amount of random environmental variability reached 17.93 thus a bit more than twice the width of an environmental niche in our model, and that amount of environmental variations only had to apply from three generations to an average of 11 generations. Again, these theoretical predictions agree with what has been observed in natural populations exposed to huge environmental changes for a short number of generations [71–73].

### Comparison with previous models

Our model combines two features of sex: recombination and fitness cost compared to clonal reproduction. [20, 56] mentioned that recombination alone is favored as soon as *V*_*θ*_>2*V*_*s*_, where *V*_*θ*_ is the variance of random environmental fluctuations, and *V*_*s*_ is the total variance of fitness induced by the environment. *V*_*s*_ is calculated as: $V_{s}=\omega^{2}+\sigma_{E}^{2}$, *ω*^2^ being defined exactly as in the present model, and $\sigma_{E}^{2}$ the environmental variance as it is usually used in quantitative genetic models. In our model, phenotypic variation is determined only by genetic differences between individuals, hence $\sigma_{E}^{2}=0$ and thus *V*_*s*_ = *ω*^2^. The variance of the random environmental fluctuations is *SD*^2^ in our model. The condition of [56] then becomes $SD>\omega \sqrt{2}$. In our simulations, we used *ω* = 4, which should result in an inflection point of 5.657 according to [56]. To check the agreement of our results with this prediction, we set the advantage of clonal populations to 1.0 (hence with no advantage) in simulations with nonnegative frequency-dependent selection and we obtained an inflection value of 5.81 ± 0.11 (the isolated point on Fig 3). The confidence interval at 95% of the value we obtained by simulation ([5.60; 6.02]) includes the [56] prediction.

### Effect of temporal autocorrelation of environmental fluctuations

Inter-generational autocorrelation along the environmental fluctuations makes these environmental fluctuations more predictable, which should favor clonal reproduction. Our simulations confirmed this intuition, as shown in Fig 2. An autocorrelation of 0.6 from one generation to the next, and without any negative frequency-dependent selection, moved the inflection point from 17.94 to 18.40, increasing the amount of environmental fluctuations needed to fix sex in 50% of the populations.

In previous studies, different effects of autocorrelation of environmental fluctuations were observed. [74], and more recently [68], found a negative effect of positive autocorrelation on the advantage of sex while [55, 56] found a positive effect. Although both [56, 74] provide convincing mathematical developments, they lead to contradictory predictions, and our simulations supported the results of [68, 74]. We failed to find a clear explanation to the discrepancies between these different models, but our results suggest the idea that positive autocorrelation makes the environmental fluctuations more predictable.

### Impact of negative frequency-dependent selection

The effect of negative frequency-dependent selection, as a proxy to Red Queen co-evolution between biological antagonists, was simulated very crudely in our model. It ignored, for example, the gene-for-gene or matching-allele interactions between parasites and their hosts, and we also did not model the potential evolution of parasites with its temporal feedback. This contrasts with more sophisticated models conceived to show how negative frequency-dependent selection could emerge from genetic processes [24, 31, 33, 75]. With these simplifications, negative frequency-dependent selection appeared to lower the threshold of random environmental fluctuations above which sex began to fix within populations. The negative frequency-dependent selection we used is actually both strong and linear, with mortality proportional to phenotypic frequency. In such system, should a population become monomorphic (with a single phenotypic class), its probability of death would reach 1.0 and population would immediately go extinct. Our results seem to indicate that such a negative frequency-dependent selection acts as a factor modifying the selection process imposed by random environmental fluctuations rather than as the main cause of the advantage of sex.

### Cost of sexuality

We found that any reduction in the two-fold cost of sex decreased the random environmental fluctuations needed to fix sex in populations. The accumulation of epistatic deleterious mutations seems too slow in experimental studies with different species to alone favor an actual reduction of the two-fold cost of sex [43, 75–77]. However, a diminution of the two-fold cost of sex is also expected in a *k* selection context as soon as the whole population approaches the carrying capacity of the environment [23]. Indeed, in this case, a two-fold advantage in birth rate would have low or even no differential effect, as the offspring will experience strong competition with other individuals and even with their own kinship.

### Combined effects

In the real world, all the factors discussed above likely interact simultaneously. Each of them, taken separately, led to detailed and convincing theoretical models demonstrating that they are sufficient in themselves to fix sex in populations. The present study addresses the combination of these factors including randomly fluctuating selection. We found that negative frequency-dependent selection and factors limiting the cost of sex lower the amplitude of random environmental fluctuations needed to fix sex in populations. These two results argue that the advantage of recombination imposed by sex would occur in biological and environmental contexts favoring genetic variance in progeny (*i*.*e*., the spreading of adaptive potential) rather than favoring the production of one optimal genotype as expected under directional selection. This phenomenon was initially suggested by [78] and is closely related to bet-hedging [26, 79]. Some later works, based mainly on the concept of quasi-species in viruses, cancer cells, immune system and prebiotic self-reproducing molecules, note clear evidence of selection favoring the “survival of the flattest” (*i*.*e*., competition being won by variants less specialized and with a flatter and larger distribution of traits than very sharply adapted ones; [80, 81]). This would be the key success of sexual organisms when facing uncertainty, underlying the multiple advantages of sex when facing biotic and abiotic heterogeneous environments [68].

Interestingly, experimental evolution and field studies found that heterogeneous environments favor sexual populations while stable conditions favor clonal populations. For example, experimental evolution of populations of rotifers, mixing sexual and clonal lines in controlled environments showed that heterogeneous environments in temperature, salinity and metal concentrations favor the emergence of higher proportions of sexual lines and even fixation of sexual reproduction over the generations [72]. Also, after a major change of coastal environments due to earthquake (coastal uplift and soil compositions), only sexual populations of *Agarophyton chilense*, a costal alga, locally survived these environmental changes and all clonal populations collapsed in less than two generations while such clonal lines dominate undisturbed populations [73]. Likewise, natural populations of clonal and sexual snail lineages, cured of their parasites and moved into large, stable mesocosms, increase in frequency of clonal in all the four replicates of a common garden experiment, suggesting that more heterogeneous, natural environment may favor sexual lineages against clonal advantages [71]. Overall, exclusive clonal species were found associated with both biotically and abiotically homogeneous environments, while exclusively sexual species in the same clades all develop in more heterogeneous environments [82].

Finally, the three main factors (random environmental fluctuations, negative frequency-dependent selection (as a proxy to Red Queen dynamics), and the reduction of the two-fold cost of sex either due to an overall synergistic epistatic negative effect of mutation accumulation, or because differences in fitness between sexuals and clonals are tempered by other fitness components like intra-specific competition) assumed here to explain the maintenance and fixation of sex rely all on actual and important biological phenomena that are likely to act in combination. Their combination is remarkably linear and additive on the scale of measurements we used here. They seem theoretically sufficient, when considered together, to help explaining why sex may have been fixed in so many eukaryote species, despite its fundamental cost in environments that are stable over time. Our results call for future field and experimental studies to explore the joint, pluralistic, effects of such combinations of processes including fluctuating selection.
